# Susceptibility of Ugandan Plasmodium falciparum Isolates to the Antimalarial Drug Pipeline

**DOI:** 10.1128/spectrum.05236-22

**Published:** 2023-05-09

**Authors:** Oriana Kreutzfeld, Patrick K. Tumwebaze, Martin Okitwi, Stephen Orena, Oswald Byaruhanga, Thomas Katairo, Melissa D. Conrad, Stephanie A. Rasmussen, Jennifer Legac, Ozkan Aydemir, David Giesbrecht, Barbara Forte, Peter Campbell, Alasdair Smith, Hiroki Kano, Samuel L. Nsobya, Benjamin Blasco, Maelle Duffey, Jeffrey A. Bailey, Roland A. Cooper, Philip J. Rosenthal

**Affiliations:** a University of California, San Francisco, San Francisco, California, USA; b Infectious Diseases Research Collaboration, Kampala, Uganda; c Dominican University of California, San Rafael, California, USA; d Program in Molecular Medicine, University of Massachusetts Medical School, Worcester, Massachusetts, USA; e Brown University, Providence, Rhode Island, USA; f Wellcome Centre for Anti-Infectives Research, Division of Biological Chemistry and Drug Discovery, University of Dundee, Dundee, United Kingdom; g Mitsubishi Tanabe Pharma Corporation, Yokohama, Japan; h Medicines for Malaria Venture, Geneva, Switzerland; University of Virginia

**Keywords:** *Plasmodium falciparum*, Ugandan field isolates, antimalarials, drug resistance, genotypic identification, malaria, susceptibility testing

## Abstract

Malaria, especially Plasmodium falciparum infection, remains an enormous problem, and its treatment and control are seriously challenged by drug resistance. New antimalarial drugs are needed. To characterize the Medicines for Malaria Venture pipeline of antimalarials under development, we assessed the *ex vivo* drug susceptibilities to 19 compounds targeting or potentially impacted by mutations in P. falciparum ABC transporter I family member 1, acetyl-CoA synthetase, cytochrome *b*, dihydroorotate dehydrogenase, elongation factor 2, lysyl-tRNA synthetase, phenylalanyl-tRNA synthetase, plasmepsin X, prodrug activation and resistance esterase, and V-type H^+^ ATPase of 998 fresh P. falciparum clinical isolates collected in eastern Uganda from 2015 to 2022. Drug susceptibilities were assessed by 72-h growth inhibition (half-maximum inhibitory concentration [IC_50_]) assays using SYBR green. Field isolates were highly susceptible to lead antimalarials, with low- to midnanomolar median IC_50_s, near values previously reported for laboratory strains, for all tested compounds. However, outliers with decreased susceptibilities were identified. Positive correlations between IC_50_ results were seen for compounds with shared targets. We sequenced genes encoding presumed targets to characterize sequence diversity, search for polymorphisms previously selected with *in vitro* drug pressure, and determine genotype-phenotype associations. We identified many polymorphisms in target genes, generally in <10% of isolates, but none were those previously selected *in vitro* with drug pressure, and none were associated with significantly decreased *ex vivo* drug susceptibility. Overall, Ugandan P. falciparum isolates were highly susceptible to 19 compounds under development as next-generation antimalarials, consistent with a lack of preexisting or novel resistance-conferring mutations in circulating Ugandan parasites.

**IMPORTANCE** Drug resistance necessitates the development of new antimalarial drugs. It is important to assess the activities of compounds under development against parasites now causing disease in Africa, where most malaria cases occur, and to determine if mutations in these parasites may limit the efficacies of new agents. We found that African isolates were generally highly susceptible to the 19 studied lead antimalarials. Sequencing of the presumed drug targets identified multiple mutations in these genes, but these mutations were generally not associated with decreased antimalarial activity. These results offer confidence that the activities of the tested antimalarial compounds now under development will not be limited by preexisting resistance-mediating mutations in African malaria parasites.

## INTRODUCTION

Malaria, especially Plasmodium falciparum infection, remains a major problem, particularly in Africa, and its treatment and control are seriously challenged by drug resistance (1). With widespread resistance to older agents, artemisinin-based combination therapies (ACTs) are the mainstays for the treatment of falciparum malaria. However, ACT efficacy is threatened by resistance to artemisinins, which is established in Southeast Asia and has recently emerged in East Africa (2–4). In addition, resistance to key artemisinin partner drugs has emerged in Southeast Asia, associated with poor treatment efficacy of ACTs (5). New antimalarial drugs are urgently needed.

Medicines for Malaria Venture (MMV) was established to discover and develop new antimalarials in partnership with the pharmaceutical industry and academic researchers (6–8). Drugs are needed to treat active malaria infections by killing asexual erythrocytic parasites, prevent malaria by killing liver-stage and erythrocytic parasites, and block malaria transmission by acting against sexual-stage parasites (7). MMV has maintained a pipeline of compounds under discovery and development as potential new antimalarial agents over the last 2 decades (8). During discovery and development, these compounds are assessed for activity against cultured malaria parasites, activity against different life cycle stages, ease of *in vitro* resistance selection, and drug-like features, including pharmacokinetics, chemical and environmental stability, susceptibility to metabolism, toxicity, and safety (9).

An important goal is to ensure that resistance to newly developed antimalarial drugs is unlikely to emerge (10, 11). A key concern is whether varied susceptibilities to new compounds under development are present in malaria parasites now circulating in regions where malaria is endemic. Thus, it is important to determine the drug susceptibilities and genotypic profiles of malaria parasites freshly isolated in malaria-endemic regions where malaria is endemic (10, 11). We previously evaluated the *ex vivo* activities of inhibitors of P. falciparum Na^+^ ATPase 4 (PfATP4) (12), dihydrofolate reductase (PfDHFR) (13), and proteasome components (14) against cultured Ugandan P. falciparum isolates and determined the genetic diversity of the targets of these inhibitors. Ugandan isolates were universally highly sensitive to these lead inhibitors, but modest changes in compound susceptibility were associated with genetic polymorphisms in genes encoding PfATP4, PfDHFR, and the proteasome β2 subunit (12–14).

To more broadly assess the susceptibilities of African parasites to compounds in the MMV development pipeline, we determined the *ex vivo* susceptibilities of 998 fresh P. falciparum clinical isolates collected in eastern Uganda to 19 compounds representing diverse chemical classes and acting against or impacted by 11 different target proteins. We also sequenced these predicted targets to assess genetic variation, gain insights into potential mechanisms mediating varied susceptibilities, and allow genotype-phenotype association studies.

## RESULTS

### Compounds under study.

We evaluated 19 compounds now under study or in development at MMV as potential new antimalarials (Table 1). These included compounds targeting or potentially impacted by mutations in P. falciparum ATP-binding cassette transporter I family member 1 (PfABCI3; encoded by PF3D7_0319700), acetyl-CoA synthetase (PfAcAS; PF3D7_0627800), cytochrome b (PfCYTB; Pf3D7_MIT02300, mal_mito_3), dihydroorotate dehydrogenase (PfDHODH; PF3D7_0603300), elongation factor 2 (PfeEF2; PF3D7_1451100), lysyl-tRNA synthetase (PfKRS; PF3D7_1350100), phenylalanyl-tRNA ligase alpha subunit (PfFRS; PF3D7_0109800), plasmepsin X (PfPMX; PF3D7_0808200), prodrug activation and resistance esterase (PfPARE; PF3D7_0709700), and the V-type H+ ATPase subunit D (PfVATPase-D; PF3D7_1341900). The compounds were studied for *ex vivo* activity against freshly cultured P. falciparum isolates collected in eastern Uganda from 2015 to 2022, potential resistance mediators for each compound were sequenced, and genotype-phenotype associations were investigated.

**TABLE 1:** *Ex vivo* susceptibilities of Ugandan P. falciparum isolates to MMV pipeline antimalarials

Putative target or resistance mechanism (P. falciparum)	Compound	Partner^*a*^	Predicted target function	Phase of discovery/development	Time period of testing	No. of isolates^*b*^	Median IC_50_ (nM)			
							*Ex vivo*			*In vitro* ^*d*^
							Ugandan field isolates	3D7^*c*^	Dd2^*c*^	
ABCI3	MMV892977 (CC0998453)	Celgene	ATP-binding cassette transporter	Discovery compound/halted	July 2017–Sept 2020	138	24.5	26.7	92.3	50

AcAS	MMV693183	TropIQ	Combines acetate and CoA to form acetyl-CoA	Candidate surveillance	Feb 2019–May 2022	441	1.8	3.0	2.1	5
	MMV689258	TropIQ		Discovery compound	July 2017–Sept 2020	129	4.6	8.0	11.1	5
	MMV-MTPC_01	MTPC		Discovery compound	Dec 2018–July 2019	89	5.3	6.1	3.9	5
	MMV-MTPC_02	MTPC		Discovery compound	Oct 2020–Jan 2021	47	3.0	3.0	1.5	NA

CYTB	MMV034055 (ELQ300)	OHSU/MMV	Reoxidizes coenzyme Q following its reduction into the parasite mitochondrion	Preclinical	Jan 2017–May 2022	631	13.0	17.8	18.3	20

DHODH	MMV669059 (DSM421)	UTSW	*De novo* pyrimidine biosynthesis	Candidate surveillance/halted	Dec 2015–Sept 2020	555	20.0	14.5	14.7	50
	MMV018912 (DSM265)	UTSW/Takeda		Phase II/halted	Dec 2015–Sept 2020	486	3.6	2.2	2.6	5
	MMV897680 (DSM632)	UTSW		Discovery compound	July 2017–Sept 2020	416	9.8	7.0	7.9	5
	MMV689256 (BRD1331)	Broad Institute/Eisai		Discovery compound	July 2017–Sept 2020	462	14.0	5.1	5.4	50
	MMV1545632 (DSM705)	UTSW		Discovery compound	May 2018–Sept 2020	184	11.0	6.0	6.1	10
	MMV1803150 (DSM1049)	UTSW		Discovery compound	Oct 2020–Aug 2021	137	22.0	16.1	15.2	15

eEF2	MMV643121 (DDD498/M5717)	Merck KGaA	GTP-dependent translocation of the ribosome along mRNA	Phase I	Jan 2017–May 2022	397	0.6	0.3	0.3	1

KRS	MMV1633780	Dundee	Lysyl-tRNA synthetase	Discovery compound	June 2019–Sept 2020	142	1.1	0.8	0.8	3

FRS	MMV692817 (BRD5018)	Broad Institute/Eisai	Phenylalanyl-tRNA synthetase	Discovery compound	July 2017–Sept 2020	346	1.6	1.2	1.9	5

PMX	MMV1782317 (compound 4)	UCB	Aspartic protease involved in erythrocyte egress and invasion	Discovery compound	Aug 2019–Sept 2020	99	12.0	10.5	18.5	NA
	MMV1794913 (UCB7362)	UCB		Discovery compound	Oct 2020–Jan 2021	39	12.0	8.5	16.0	15

PARE	MMV675867 (AN13762)	Anacor	Activation of antimalarial esters and other compounds	Surveillance	Jan 2017–May 2022	709	71.0	42.9	64.0	100

VATPase-D	MMV674253 (AZ412/ZY19489)	Zydus	V-type H^+^-ATPase; extrudes protons from parasites	Surveillance	Aug 2015–May 2022	760	3.4	2.7	3.9	10

a Anacor, Anacor Pharmaceuticals Inc., Palo Alto, CA; Broad Institute, Broad Institute, Cambridge, MA; Celgene, Celgene Global Health, Summit, NJ; Dundee, University of Dundee, Dundee, United Kingdom; Eisai, Eisai Co. Ltd., Tokyo, Japan; Merck KGaA, the health care business of Merck KGaA, Darmstadt, Germany; MTPC, Mitsubishi Tanabe Pharma Corporation, Yokohama, Japan; UCB, UCB Biopharma SPRL SA, Slough, United Kingdom; OHSU, Oregon Health and Science University, Portland, OR; UTSW, the University of Texas Southwestern Medical Center, Dallas, TX; TropIQ, TropIQ Health Sciences, Nijmegen, The Netherlands; Zydus, Zydus Pharmaceuticals, Ahmedabad, India.

b Number of Ugandan isolates studied in *ex vivo* susceptibility assays.

c The 3D7 and Dd2 control strains were tested monthly.

d IC_50_s from previous *in vitro* studies with laboratory strains. NA, not applicable.

### Activities of the tested antimalarials against Ugandan isolates.

**FIG 1:**
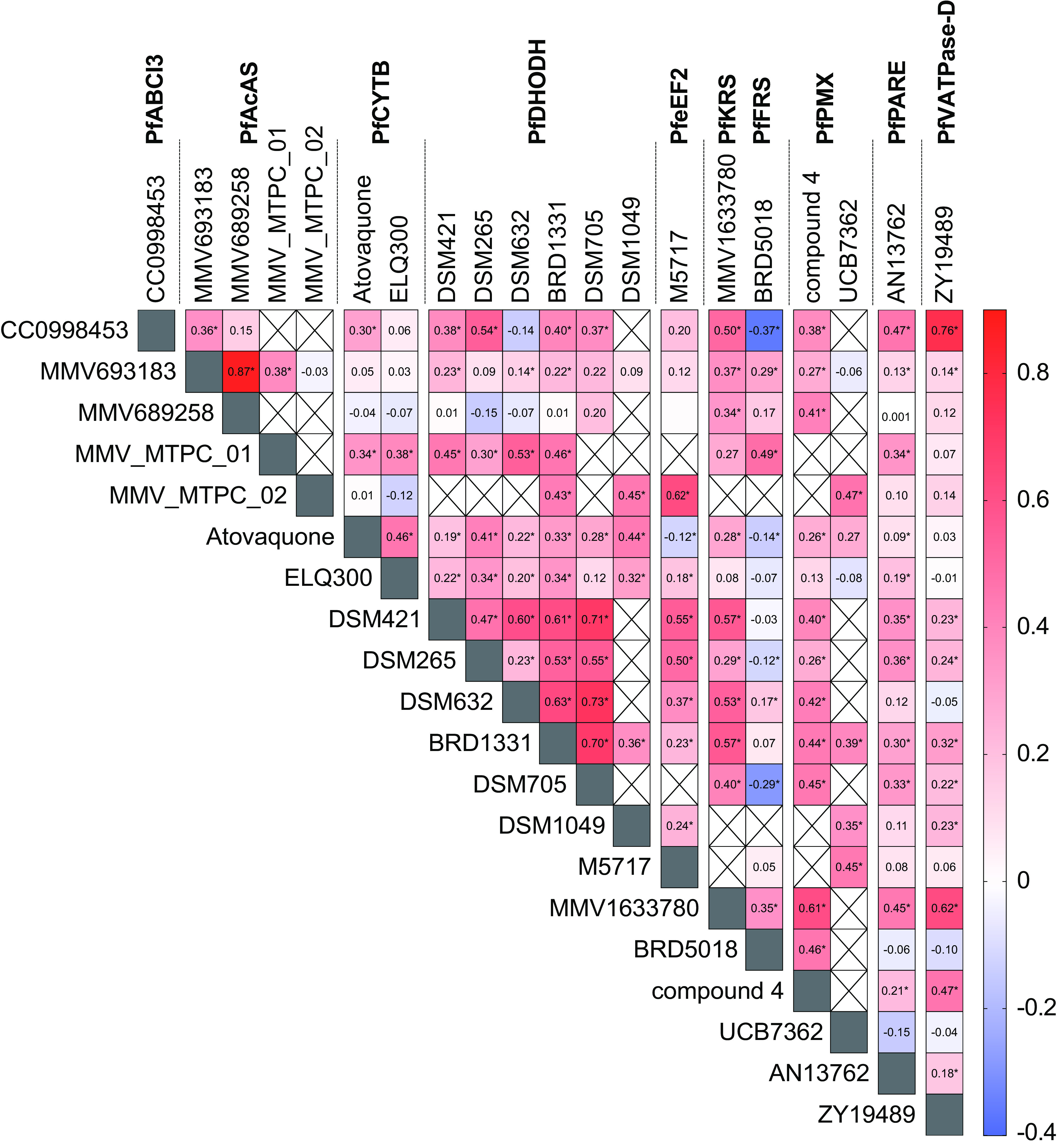
Correlations between activities of tested antimalarials. The heat map demonstrates correlations between the IC_50_s for individual isolates for all studied inhibitors. The inhibitors are organized based on predicted targets, which are indicated at the top. Spearman’s coefficients are indicated numerically and by the color scale, with higher positive values indicating stronger correlations. Associations that were statistically significant (*P < *0.05) are indicated by an asterisk. Correlations that could not be determined due to the testing of compounds during different time frames are indicated with an X.

Half-maximum inhibitory concentrations (IC_50_s) were determined for 998 fresh Ugandan isolates using a SYBR green assay. Overall, the field isolates were highly susceptible to lead antimalarials, with low- to midnanomolar median IC_50_s, near values previously reported for laboratory strains, for all 19 tested compounds, although outliers with decreased susceptibilities were identified (Table 1). We assessed correlations of the IC_50_s of individual isolates for different compounds. For compounds with the same predicted target, strong positive correlations were generally seen (Spearman rank *r* coefficient of >0.6), suggesting that similar modes of action are driving susceptibility to the different compounds (Fig. 1). Interestingly, we observed strong correlations between compounds with potential modes of resistance involving PfVATPase-D (ZY19489) and PfABCI3 (CC0998453) and both positive and negative correlations between inhibitors of PfAcAS (MMV693183, MMV689258, MMV-MTPC_01, and MMV-MTPC_02).

### PfABCI3.

**FIG 2:**
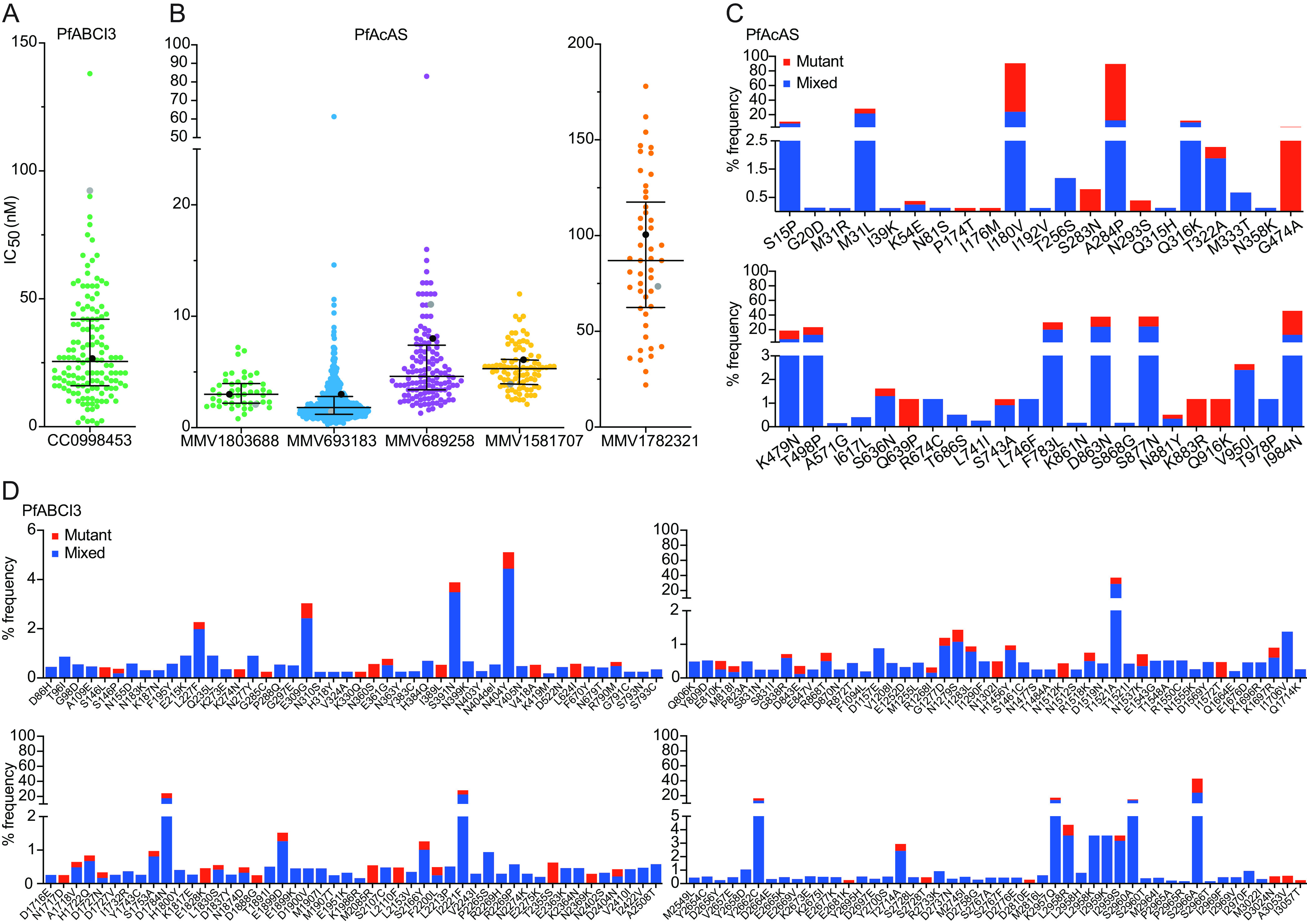
PfABCI3 and PfAcAS inhibitor susceptibilities and genotypes of Ugandan P. falciparum isolates. (A and B) *Ex vivo* susceptibilities of Ugandan isolates to PfABCI3 (A) and PfAcAS (B) inhibitors. Each dot depicts a single isolate, except that median IC_50_s for 3D7 (black) and Dd2 (gray) are depicted as single dots. Horizontal lines show median IC_50_s and interquartile ranges. (C and D) Frequencies of PfAcAS (C) and PfABCI3 (D) mutations in Ugandan field isolates. Percentages of isolates with mixed (WT and mutant; blue) and pure mutant (red) genotypes are shown.

PfABCI3 is a P. falciparum protein belonging to the ABC transporter family, which includes multidrug-resistance-associated protein 1 (PfMRP1). The ABC transporters are typically involved in drug efflux (15). PfABCI3 was reported to be both a drug target and a resistance mediator (16). Isolates were generally highly susceptible to CC0998453, a compound with a putative mechanism of resistance involving PfABCI3, but several isolates had markedly decreased susceptibility compared to the median IC_50_ (Fig. 2A and Table 1). The susceptibility of the 3D7 reference strain was similar to the median value for Ugandan isolates, but the Dd2 strain had an IC_50_ 4-fold higher than that for Ugandan isolates (Table 1). Sequencing revealed that *pfabci3* was highly polymorphic, but most observed mutations were detected in <1% of isolates (Fig. 2D). One mutation, S2966A, was seen commonly (24% of samples with mixed and 19% with pure mutant genotypes) and was associated with increased susceptibility of Ugandan isolates (IC_50_s of 33.1 nM for wild-type [WT], 22.9 nM for mixed, and 15.9 nM for mutant genotypes [*P < *0.001 for WT versus mutant genotypes]) to CC0998453; other identified mutations were not associated with changes in *ex vivo* susceptibility (see Table S1 in the supplemental material). Additionally, a deletion of 12 amino acids (RNEKNEKNGKNE; R344 to G355) was associated with increased susceptibility to CC0998453 (IC_50_s of 43.6 nM for WT, 25.0 nM for mixed, and 26.4 nM for mutant genotypes [*P *= 0.02 for WT versus mixed genotypes; *P *= 0.06 for WT versus mutant genotypes]) (Fig. S1A and Table S1).

### PfAcAS inhibitors.

PfAcAS is responsible for converting acetate and coenzyme A to acetyl-CoA (17). We studied two pantothenamide inhibitors, MMV693183 and MMV689258, which were previously shown to have low-nanomolar activity against Ugandan P. falciparum isolates (18, 19), and two additional inhibitors, MMV-MTPC_01 and MMV-MTPC_02. In previous studies, MMV693183 reduced acetyl-CoA levels in blood-stage malaria parasites, and mutations in PfAcAS were associated with decreased activity against laboratory strains (18). Both inhibitors are active in blood stages and block transmission to *Anopheles* mosquitoes (18, 19). *Ex vivo* assays showed median IC_50_s for the 4 tested inhibitors in the low- to midnanomolar range (Fig. 2B and Table 1). A small number of isolates had IC_50_s ≥3-fold higher than the median values for MMV693183 and MMV689258 (Fig. 2B). Sequencing of Ugandan P. falciparum isolates revealed that *pfacas* was highly polymorphic, with 43 different nonsynonymous single nucleotide polymorphisms (SNPs) identified and ~40% of isolates with various numbers of asparagines, compared to the 3D7 reference strain, in a stretch beginning at codon 393 (Fig. 2C and Fig. S1B). Previous studies showed that pantothenamides act against PfAcAS but that acetyl-CoA synthetase 11 (PfACS11; PF3D7_1238800) (Fig. S2) might play a role in resistance (18–20). One mutation in PfACS11, I183M, was associated with increased susceptibility to MMV693183 in isolates with mixed genotypes (IC_50_s of 2.8 nM for WT, 1.4 nM for mixed, and 2.2 nM for mutant genotypes [*P* = 0.04 for WT versus mixed]) (Table S1). However, none of the observed polymorphisms were associated with decreased susceptibility to any of the studied PfAcAS inhibitors.

### PfCYTB inhibitors.

**FIG 3:**
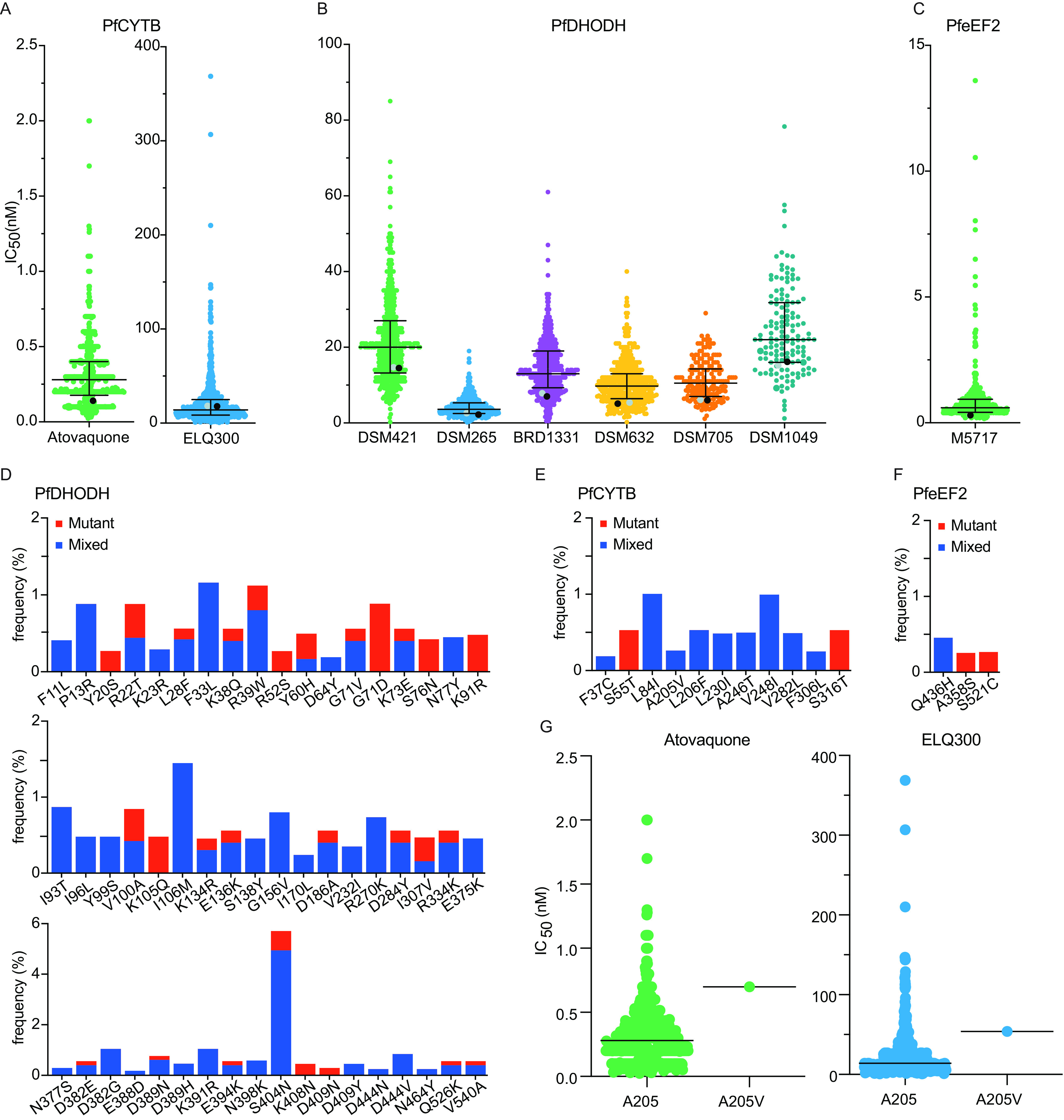
PfCYTB, PfDHODH, and PfeEF2 inhibitor susceptibilities and genotypes of Ugandan P. falciparum isolates. (A to C) *Ex vivo* susceptibilities of Ugandan isolates to inhibitors of PfCYTB (A), PfDHODH (B), and PfeEF2 (C). (D to F) Frequencies of PfCYTB (E), PfDHODH (D), and PfeEF2 (F) mutations in Ugandan field isolates. (G) *Ex vivo* IC_50_s of parasites with either the PfCYTB WT (A205) or mixed (A205V) genotype at position 205. The formatting and labeling are the same as those described in the legend of Fig. 2.

PfCYTB sits in the mitochondrial membrane, where it is part of respiratory chain complex III, which is responsible for electron transport. ELQ300, an endochin-like quinolone, targets PfCYTB in the liver, blood, and transmission stages, as does the approved antimalarial atovaquone (21). Isolates were highly susceptible to atovaquone and ELQ300 (Fig. 3A and Table 1). Some isolates had IC_50_s ≥3-fold above the median value. Eleven PfCYTB mutations were detected, each in ≤3 isolates (Fig. 3E). A single isolate with the A205V mutation had susceptibilities to both atovaquone and ELQ300 well above the median values (Fig. 3G).

### PfDHODH inhibitors.

PfDHODH is required by P. falciparum for pyrimidine biosynthesis in erythrocytic and liver stages. We studied a series including 2 triazolopyrimidine-based, 2 pyrrole-based, and 2 related inhibitors targeting PfDHODH (22–26). The isolates had various susceptibilities to these 6 inhibitors (Fig. 3B and Table 1), although only a small minority had IC_50_s 3-fold higher than the median values. Sequencing of Ugandan P. falciparum isolates revealed a large number of mutations in *pfdhodh*, most of which were detected in <1% of samples (Fig. 3D). None of the observed mutations were among those previously selected in laboratory strains with *in vitro* drug pressure (24, 27, 28) or associated with decreased *ex vivo* susceptibility to any of the tested PfDHODH inhibitors.

### PfeEF2 inhibitor.

PfeEF2 mediates the translocation of the ribosome along mRNA. M5717, a quinolone-4-carboxamide, targets PfeEF2 in multiple life cycle stages (29–31). Ugandan isolates were highly susceptible to M5717 (Fig. 3C). Only three mutations were detected in PfeEF2 (Q436H, A358S, and S521C), each in one isolate (Fig. 3F). None of the observed mutations were associated with decreased susceptibility to M5717.

### tRNA synthetase inhibitors.

**FIG 4:**
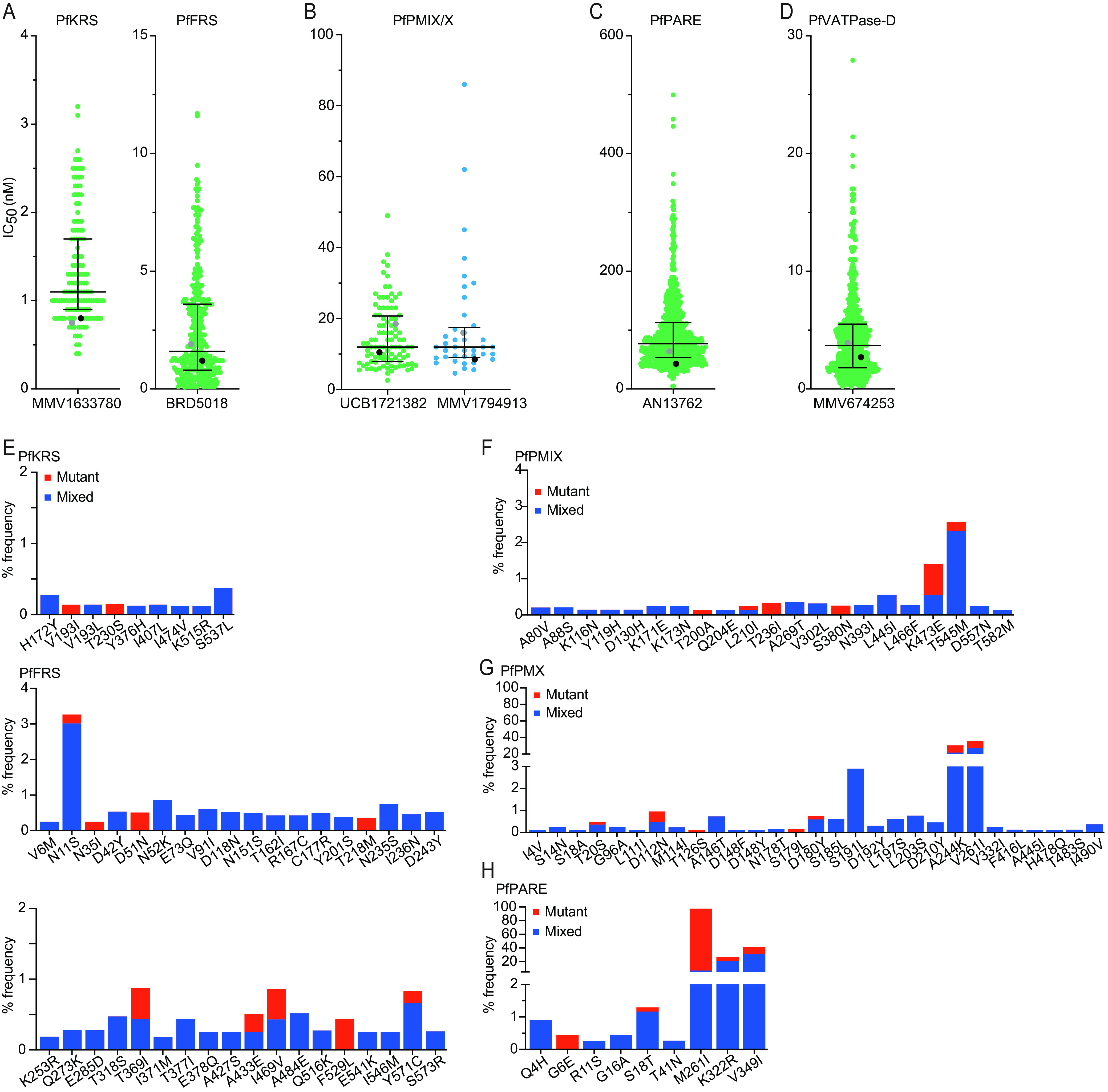
PfKRS/FRS, PfPMX, PfPARE, and PfVATPase-D inhibitor susceptibilities and genotypes of Ugandan P. falciparum isolates. (A to D) *Ex vivo* susceptibilities of Ugandan isolates to inhibitors of or interactors with PfKRS and PfFRS (A), PfPMX (B), PfPARE (C), and PfVATPase-D (D). (E to H) Frequencies of PfKRS and PfFRS (E), PfPMIX (F), PfPMX (G), and PfPARE (H) mutations in Ugandan field isolates. The formatting and labeling are the same as those described in the legend of Fig. 2.

Multiple inhibitors of tRNA synthetases, which are required for the aminoacylation of tRNAs, have shown potent antimalarial activity (32–35). MMV1633780 inhibits PfKRS in blood and liver stages (36–38), and the bicyclic azetidine BRD5018 inhibits PfFRS in the blood and liver stages and prevents transmission to *Anopheles* mosquitoes (32, 34). Isolates showed susceptibilities to both MMV1633780 and BRD5018 in the low-nanomolar range (Fig. 4A and Table 1). The median IC_50_ of MMV1633780 was 2-fold higher than those for the control strains 3D7 and Dd2 (Fig. 4A and Table 1). Sequencing of field isolates revealed 9 mutations in PfKRS, each detected in only 1 isolate, except that 3 isolates had an S537L mutation (Fig. 4E). Several mutations were identified in PfFRS (Fig. 4E), among which was N11S, which was present in 26 isolates. Many insertions and deletions were observed in asparagine-rich regions of PfFRS (Fig. S1C). None of the observed mutations were associated with decreased susceptibility to MMV1633780 or BRD5018.

### PfPMX inhibitors.

PfPMX is found in merozoite exonemes, and the inhibition of this aspartic protease leads to the inhibition of erythrocyte invasion (39–41). Ugandan isolates were highly susceptible to 2 tested cyclic acyl guanidine inhibitors, compound 4 and UCB7362 (42), with some variation in susceptibilities (Fig. 4B and Table 1). Sequencing revealed that *pfpmx* was polymorphic, but mutations were found in <1% of samples, except for V261I, which was present in 36%, and R244K, which was present in 31% of the isolates (Fig. 4F). These two mutations were associated with modest increases in susceptibility to compound 4 (V261I, 21.5 nM for WT, 13.8 nM for mixed, and 12.6 nM for mutant genotypes [*P *= 0.04]; R244K, 21.7 nM for WT, 12.8 nM for mixed, and 12.6 nM for mutant genotypes [*P *= 0.03]) (Table S1) but not UCB7362. Multiple insertions and deletions were detected between amino acids 184 and 205 of PfPMX (Fig. S1D). Two stretches of deletions were associated with an increase (S185 to N196 deletion, 22.2 nM for WT and 13.1 nM for mixed genotypes [*P < *0.001]) or a decrease (L203 to N208 deletion, 14.4 nM for WT and 202 nM for mixed genotypes [*P *= 0.03]) in susceptibility, but the analysis was limited by the absence of pure mutant samples (Table S1). We additionally looked at two other plasmepsins, PfPMV (PF3D7_1323500), which plays a role in protein export in erythrocytic stages, and PfPMIX (PF3D7_1430200), found in rhoptry secretory organelles. Previous studies looked at the potency of PfPMX inhibitors against other plasmepsins; both PfPMV and PfPMIX were only slightly inhibited by these compounds (42). Sequencing revealed many mutations in *PfPMV* and *PfPMIX*, each in <3% of the isolates (Fig. S2), but none were associated with changes in susceptibility to UCB7362 or compound 4.

### PfPARE.

PfPARE is a parasite esterase that activates some antimalarial compounds (43). Loss-of-function mutations in PfPARE were associated with resistance to pepstatin esters, MMV011438, and AN13762 (43, 44). Ugandan isolates showed various susceptibilities to AN13762 (Fig. 4C and Table 1). Sequencing revealed 9 mutations in PfPARE, 3 of them at high prevalences (Fig. 4G); none were nonsense mutations, which were readily selected *in vitro*. The V349I mutation (present in 41% of the isolates) was associated with slightly decreased susceptibility to AN13762 (IC_50_s of 68.5 nM for WT, 77.8 nM for mixed, and 71.9 nM for mutant genotypes [*P *= 0.01 for WT versus mixed genotypes; *P *= 0.56 for WT versus mutant genotypes]) (Table S1). No mutations were seen in SUMO-activating enzyme subunit 2 (PfUBA2; PF3D7_123700) an enzyme for which mutations were previously seen in parasites selected *in vitro* for high-level resistance to AN13762 (43).

### PfVATPase-D.

PfVATPase-D is a proton pump that enables the acidification of the P. falciparum digestive vacuole (45). *In vitro* resistance to the triaminopyrimidine blood-stage inhibitor ZY19489 was difficult to achieve, but parasites with a 3- to 6-fold increase in the IC_50_ had a mutation in PfVATPase-D (46). Ugandan isolates showed various susceptibilities to ZY19489 (Fig. 4D and Table 1). Sequencing revealed no mutations in *pfVATPase-D* in any Ugandan isolate.

## DISCUSSION

MMV and its partners are evaluating multiple compounds for development as potential new antimalarials. As part of this evaluation, it is important to characterize susceptibilities to lead compounds and the genetic diversity of compound targets in parasites now circulating in Africa (11). We tested 19 compounds from the MMV antimalarial pipeline against fresh Ugandan clinical isolates, sequenced the genes encoding the predicted targets of these compounds, and searched for genotype-phenotype associations. All compounds showed activity in the low- to midnanomolar range, suggesting appropriate potency for effective therapeutic efficacy. Some variation in activity was seen for every tested compound, but definitive decreases in susceptibility were not associated with specific genotypes. Unexpectedly, a few polymorphisms were associated with increased drug susceptibility. Our work offers insight into the susceptibilities of African parasites to next-generation antimalarials and the genetic diversity of the predicted targets of these compounds. Overall, Ugandan parasites were highly susceptible to MMV pipeline drugs, and phenotypic and genomic results did not suggest preexisting resistance to any of the tested compounds.

Ugandan parasites showed some variability in susceptibilities to all compounds, but significant changes, possibly associated with specific genotypes, were seen for only a small number of compounds. For three compounds, acting against PfABCI3, PfAcAS, and PfPMX, polymorphisms in Ugandan isolates were surprisingly associated with enhanced susceptibility. PfABCI3, a transporter, was highly polymorphic (189 mutations), and one point mutation (S2966A) and one deletion (positions R344 to G355) were each associated with increased susceptibility to CC0998453. Pantothenamides have been found to act against PfAcAS and PfACS11, another CoA-binding enzyme, which was suggested to be a mediator of resistance (18, 19). Interestingly, Ugandan parasites with mutation I183M in PfACS11 had increased susceptibility to MMV693183. Altered susceptibility with this mutation was not observed for any of the other tested PfAcAS inhibitors, consistent with another study that did not find altered susceptibility to another PfAcAS inhibitor, MMV019721, in parasites with PfACS11 mutations (20). PfPMX, an aspartic protease, is a target of compound 4. About one-third of Ugandan parasites carried both the V261I and R244K mutations, and both were associated with increased susceptibility compared to the wild-type sequence. Additionally, a deletion in PfPMX was associated with a slight decrease in susceptibility to MMV1782317 for mixed genotypes, but the absence of pure mutant genotypes limited further analysis. In PfPARE, three mutations, M261I, K322R, and V349I, were common, and V349I was associated with slightly decreased susceptibility in isolates with mixed genotypes compared to the wild type. Overall, some mutations identified in Ugandan parasites were associated with increases or decreases in susceptibilities to some compounds, but in all cases, differences in susceptibilities were modest, and it is unclear if, among a large number of comparisons, these results represent biologically relevant differences or only occasional differences due to chance.

The antimalarial candidate ELQ300 and the approved drug atovaquone target the mitochondrial electron transport chain. Ugandan parasites were highly susceptible to ELQ300, but there was variability in the results, with several isolates having IC_50_s 4- to 6-fold above the median IC_50_. Some of this variability may have been due to the limited solubility of ELQ300 under the assay conditions rather than truly decreased activity; new bioreversible alkoxycarbonate ester prodrugs of ELQ300, i.e., ELQ331, are under study to overcome problems with the solubility and high crystallinity of the parent compound (47). Mutations in PfCYTB were uncommon, but a single isolate with an A205V mutation had an IC_50_ 2.5-fold above the median values for both atovaquone and ELQ300. Resistance to both compounds was previously selected *in vitro*, accompanied by mutations in the PfCYTB quinol oxidase (Q_o_) site for atovaquone and the quinone reductase (Q_i_) site for ELQ300 (48, 49). A205V lies within the Q_i_ site, but with only one mutant isolate available for analysis, its role in mediating altered susceptibility is not clear. The structural characterization of other Q_i_ inhibitors showed that amino acid 205 was not involved in the binding of the 4(1H)-pyridones GSK932121 and GW844520 (50). Overall, our data suggest consistently excellent susceptibility of field isolates to ELQ300.

Six novel inhibitors from four distinct chemical series target PfDHODH. Two of these compounds, DSM265 and DSM421 (24, 25), were developed beyond the initial exploratory stage, but development was later halted, and additional inhibitors are now under study (26, 51–53). Ugandan parasites were highly susceptible to PfDHODH inhibitors, but occasional isolates had IC_50_s 3- to 4-fold higher than the median values. In clinical and *in vitro* studies, selection for resistance to PfDHODH inhibitors mediated by mutations in PfDHODH was easily achieved (22, 24, 27, 28). In Ugandan isolates, a large number of PfDHODH polymorphisms were detected, but each was identified in only a small number of isolates, none of the polymorphisms observed in these isolates were those previously selected by *in vitro* drug pressure, and none were associated with altered *ex vivo* susceptibility to the tested PfDHODH inhibitors. Thus, considering the susceptibilities of African parasites, second-generation PfDHODH inhibitors remain promising new antimalarials.

Our work offers insights into the susceptibilities of parasites now circulating in East Africa to MMV pipeline antimalarials and the genetic diversity of the targets of these compounds. Ugandan isolates were highly susceptible to all 19 tested compounds, and the genetic diversity in genes encoding target enzymes was limited. Importantly, no resistance-mediating mutations previously selected *in vitro* were present in the field isolates. Thus, none of the tested compounds appear to be challenged by preexisting drug resistance in circulating African parasites continued study of the susceptibilities of P. falciparum isolates from East Africa and other regions is needed to ensure that the MMV development pipeline is not constrained by genetic polymorphisms already present in field parasites.

## MATERIALS AND METHODS

### Samples for study.

Subjects over 6 months of age presenting between December 2015 and July 2022 at three outpatient clinics in eastern Uganda (Tororo District Hospital, Tororo District; Masafu General Hospital, Busia District; or Busiu Health Center, Mbale District) or enrolled in cohort studies in this area with clinical suspicion of malaria and a Giemsa-stained blood film positive for P. falciparum and without signs of severe disease were enrolled after informed consent was obtained, as previously described (54). Blood was collected into a heparinized tube before treatment with artemether-lumefantrine, according to national guidelines. The studies were approved by the Makerere University Research and Ethics Committee, the Uganda National Council for Science and Technology, and the University of California, San Francisco, Committee on Human Research.

### *Ex vivo* drug susceptibility assays.

Samples with parasitemias of ≥0.3% were placed into culture after the removal of the plasma and buffy coat. Aliquots of blood samples were stored on Whatman 3MM filter paper or placed into RNAlater and stored at −20°C for subsequent molecular analysis. Compounds, provided by MMV, were prepared as 10 mM stock solutions in dimethyl sulfoxide and stored at −20°C. Working solutions were prepared within 24 h of susceptibility tests and stored at 4°C. Different compounds were studied over different time frames due to limitations in compound availability and our capacity for screening.

Drug susceptibility assays were performed as previously described (54). Briefly, drugs were serially diluted 3-fold in 96-well assay plates in complete medium (RPMI 1640 medium supplemented with 25 mM HEPES, 0.2% NaHCO_3_, 0.1 mM hypoxanthine, 100 μg/mL gentamicin, and 0.5% AlbuMAX II [Invitrogen]), and parasites were added (parasitemia of 0.2% and hematocrit of 2%) to reach a volume of 200 μL. Drug-free and parasite-free controls were included. Plates were incubated for 72 h in a humidified modular incubator under 90% N_2_, 5% CO_2_, and 5% O_2_ at 37°C, and parasite growth was quantified based on SYBR green fluorescence, as previously described (54). Briefly, after 72 h, plates were frozen at −80°C, thawed, and mixed; 100 μL from each well was transferred to a black 96-well plate containing 100 μL/well SYBR green lysis buffer (20 mM Tris buffer, 5 mM EDTA, 0.008% saponin, 0.08% Triton X-100, and 0.2 μL/mL SYBR green I); plates were incubated for 1 h in the dark at room temperature; and fluorescence was measured using a FLUOstar Omega plate reader (BMG LabTech) (485-nm excitation/530-nm emission wavelengths). Dd2 and 3D7 laboratory control strains (BEI Resources) were assessed monthly. IC_50_s were derived and variability in the results was assessed as previously described (54).

### Sequencing of Ugandan P. falciparum DNA.

DNA was extracted from dried blood spots using Chelex-100. Alternatively, RNA was extracted from samples stored in RNAlater using the PureLink RNA minikit (Invitrogen) before treatment with DNase I (New England BioLabs [NEB]), and cDNA was reverse transcribed using the SuperScript IV first-stand synthesis kit (Invitrogen) according to the manufacturer’s protocol, using oligo(dT) primers.

For a subset of samples, dideoxy sequencing was performed after the genes of interest were amplified by PCR using primers designed to target these genes (see Table S2 in the supplemental material). PCR products were purified with AMPure beads (Beckman Coulter) and sequenced by standard dideoxy techniques. Sequences were analyzed using CodonCodeAligner (CodonCode Corporation), with the 3D7 sequence as a reference.

For the majority of the samples, sequences of the genes of interest were determined using molecular inversion probe (MIP) capture and deep sequencing (55, 56). The MIP panel and specific probes were designed using MIPTools software (v.0.19.12.13 [https://github.com/bailey-lab/MIPTools]) (55). Probes and primers were described in previous publications (55, 57). MIP capture, library preparation, and sequencing were performed as previously described (55). MIPTools was used to analyze raw sequencing data (55, 56). After demultiplexing, paired-end reads were joined and filtered based on the base quality score and expected length. Individual reads were discarded if the fraction of quality scores above 30 was <70%. Further processing of the samples was done using their unique molecular identifiers (UMIs). Sequences were clustered based on their UMIs to create a specific UMI consensus sequence. For samples with multiple captures, UMIs were merged. Genotypes were called for samples with at least 10 UMIs. At least 3 UMIs were required to call alternate alleles, and 2 UMIs were required to call reference alleles.

### Statistical analyses.

Associations between genotypes and drug susceptibilities were analyzed using the Wilcoxon test in R. Associations between IC_50_ values for different compounds were assessed with Spearman’s rank test using Prism version 8.4.3. The direction and magnitude of the associations between IC_50_ values for different compounds were quantified by Spearman’s rank correlation coefficient. The statistical tests were two-tailed and considered significant at a *P* value of ≤0.05.

### Data availability.

Raw sequencing reads for target genes are available in the NCBI Sequence Read Archive under BioProject accession numbers PRJNA660547 and PRJNA850445 and GenBank accession numbers OP846663 to OP846813, OP824509 to OP824618, and OP974325 to OP974400. MIPs and PCR primers used in this study are listed in Table S2 or were described in previous publications (55, 57). MIPWrangler (https://github.com/bailey-lab/MIPWrangler) and MIPTools (https://github.com/bailey-lab/MIPTools) software are available on GitHub. Additional data and stored isolates are available from the authors upon request.
