# Supplementary material for: Susceptibility of Ugandan Plasmodium falciparum Isolates to the Antimalarial Drug Pipeline
Source: Microbiol Spectr. 2023 May 9;11(3):e05236-22. doi: 10.1128/spectrum.05236-22 (PMC10269555; doi:10.1128/spectrum.05236-22)
Supplement: Supplemental file 1 — Supplemental material. Download spectrum.05236-22-s0001.pdf, PDF file, 1.5 MB [file spectrum.05236-22-s0001.pdf]

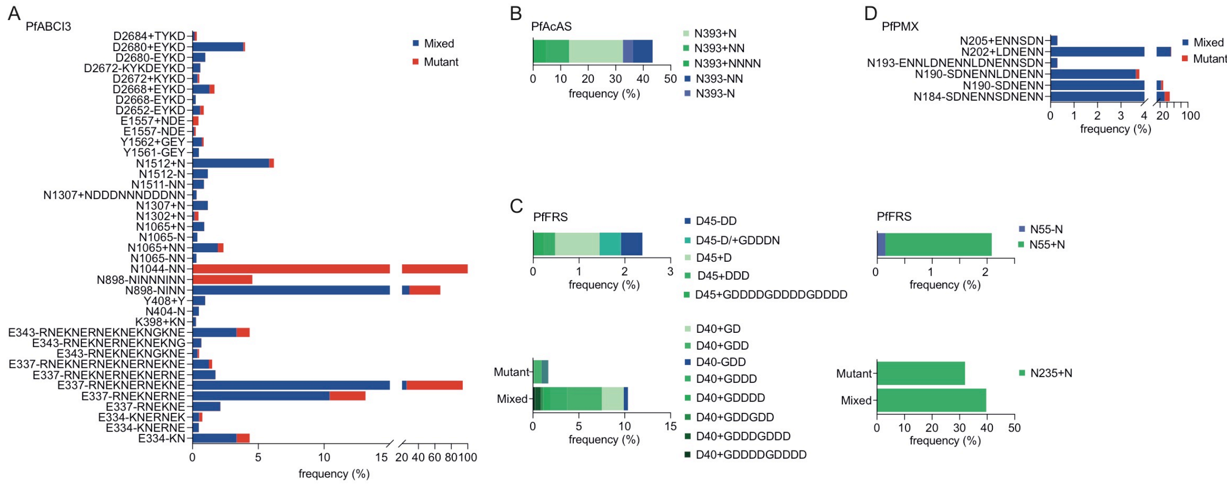

Supplementary figure 1. Insertions and deletions in putative compound targets or mediators in Ugandan field isolates. Prevalences of mixed or mutant insertions (+) and deletions (-) followed by the inserted/deleted amino acids are shown, with the color schemes defined in the keys for PfABC13 (A), PfAcAS (B), PfFRS (C), and PfPMX (D).

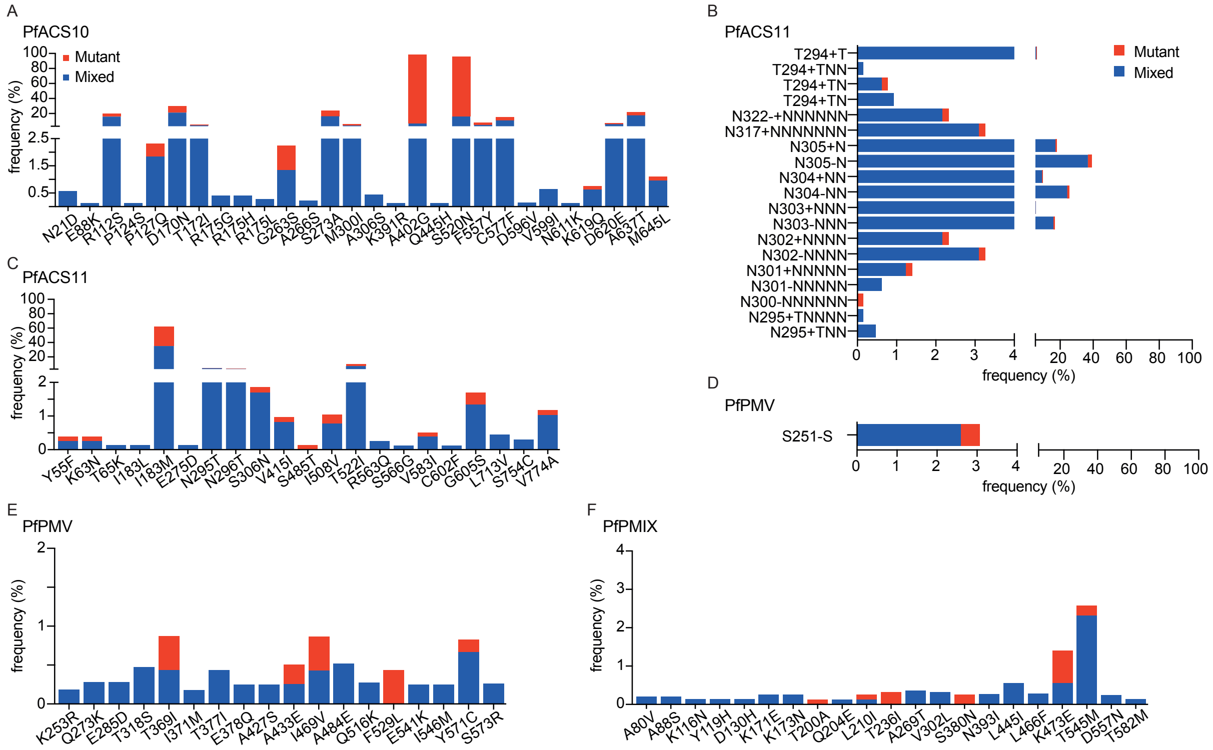

Supplementary figure 2. Genotypes of PfACS10, PfACS11, PfPMV and PfPMIX in Ugandan *P. falciparum* isolates. A,C,E+F) Frequencies of PfACS10 (A), PfACS11 (C), PfPMV (E), and PfPMIX (F) mutations in Ugandan field isolates. B+D) Insertions and deletions in PfACS11 (B) and PfPMV (D). Formatting and labeling is as in Figure 2.

Supplementary table 1. Mutations in predicted compound targets or resistance mediators associated with an increased or decreased susceptibility of Ugandan *P. falciparum* isolates

			**Median IC_{50} (nM)**				
**Compound**	**Target gene**	**Mutation**	**WT (N[a])**	**mixed (N[a])**	**mutant (N[a])**	***P*-value (WT vs. mixed)**	***P*-value (WT vs. mutant)**
CC0998453	ABCI3	S2966A	33.1 (40)	22.9 (15)	15.9 (18)	0.03	<0.001
CC0998453	ABCI3	R344-G355 deletion	43.6 (5)	25.0 (34)	26.4 (68)	0.02	0.06
MMV693183	ACS10	C577F	2.2 (19)	3.0 (3)	1.0 (2)	0.61	0.05
MMV693183	ACS11	I183M	2.8 (8)	1.4 (6)	2.2 (7)	0.04	0.75
MMV675867	Pare	V349I	68.5 (256)	77.8 (161)	71.9 (47)	0.01	0.56
MMV1782317	PMX	R244K	21.7 (43)	12.8 (14)	12.6 (5)	<0.001	0.03
MMV1782317	PMX	V261I	21.5 (42)	13.8 (18)	12.6 (5)	0.01	0.04
MMV1782317	PMX	S185-N196 deletion	22.2 (31)	13.1 (18)	-	<0.001	-
MMV1782317	PMX	L203-N208 deletion	14.4 (21)	20.2 (34)	-	0.03	-

[a] Number of isolates with specified genotype and IC_{50} data considered for the comparison.

Supplementary table 2 Primer list for PCRs and dideoxy sequencing of Ugandan *P. falciparum* isolates

Name	Gene	Function	Sequence
PfAcCS_F1_F2	PfAcAS; PF3D7_0627800	Forward PCR primer for fragment 1	ATGAATAATTTGAAGAGTTATGGAAGTC
PfAcCS_F1_R1	PfAcAS; PF3D7_0627800	Reverse PCR primer for fragment 1; sequencing primer	GCAGTACCATCTATATCTCTTCC
PfAcCS_F2_F1	PfAcAS; PF3D7_0627800	Forward PCR primer for fragment 2	GTAGATGATGATAATAATAGTAATATAC
PfAcCS_F2_R1	PfAcAS; PF3D7_0627800	Reverse PCR primer for fragment 2	TATAGTATGAAGAATATTACTACGG
PfAcCS_F2_R2	PfAcAS; PF3D7_0627800	Sequencing primer for fragment 2	TTATTTCTTAATTTCAATATGCTTTAAC
PfAcCS_F2_F2 seq	PfAcAS; PF3D7_0627800	Sequencing primer for fragment 2	ACCAGGATGTGCTAG
PfAcCS_F2_R2 seq	PfAcAS; PF3D7_0627800	Sequencing primer for fragment 2	CCTATATCTGCTACACAAC
PfAcCS_F1_R1 seq	PfAcAS; PF3D7_0627800	Sequencing primer for fragment 1	CACATATCCATAGCTACATC
PfAcCS_F1_F1 seq	PfAcAS; PF3D7_0627800	Sequencing primer for fragment 1	CAGCTAGCTTGAGCGATAG
PfAcCS_F1_R2 seq	PfAcAS; PF3D7_0627800	Sequencing primer for fragment 1	CGTTATTTACCATGTCAGTG
cytb_F1	PfCYTB; mal_mito_3	Forward PCR primer; Sequencing primer	TTCCTGATTATCCAGACGCT
cytb_R1	PfCYTB; mal_mito_3	Reverse PCR primer; sequencing primer	TGTTCCGCTCAATACTCAGA
cytb_ internal F	PfCYTB; mal_mito_3	Sequencing primer	GAGTTATTGGGGTGCAACTG
cytb_ internal R	PfCYTB; mal_mito_3	Sequencing primer	CACTCACAGTATATCCTCCACA

dhodh_ flanking_F1	PfDHODH; PF3D7_0603300	Forward PCR primer for 1st nested PCR	AAATAATAATTGAAGGGCCA
dhodh_ flanking_R1	PfDHODH; PF3D7_0603300	Reverse PCR primer for 1st nested PCR	CGACAAGTTGTTTAATAAATCCAC
dhodh_F1	PfDHODH; PF3D7_0603300	Forward PCR primer for 2nd nested PCR; sequencing primer	GTGATAGATAGCTCCAGTCGATTTC
dhodh_R1	PfDHODH; PF3D7_0603300	Reverse PCR primer for 2nd nested PCR	CATTTAAGCCCCAAAACATTTTTAC
dhodh_F2	PfDHODH; PF3D7_0603300	Sequencing primer	GCTATTAATGTAAGCTCCCC
dhodh_F3	PfDHODH; PF3D7_0603300	Sequencing primer	CCATTCGGTGTTGCTGC
dhodh_R2	PfDHODH; PF3D7_0603300	Sequencing primer	TTTGCGCACTTATGTGTCGCCC
dhodh_R3	PfDHODH; PF3D7_0603300	Sequencing primer	GCATTACCCGTTTGGCCCCTTGGGG
eEF2_F1	PfeEF2; PF3D7_1451100	Forward PCR primer for 1st and 2nd nested PCR; sequencing primer	CTGATAGCAAAATGGTGAAC
eEF2_R1	PfeEF2; PF3D7_1451100	Reverse PCR primer for 1st nested PCR	TTTCTTCTTTAATATGTGGTGC
eEF2_R2	PfeEF2; PF3D7_1451100	Reverse PCR primer for 2nd nested PCR; sequencing primer	TACATATTTGTTGTAGTAGTGG
eEF2_F2	PfeEF2; PF3D7_1451100	Sequencing primer	AACCTTTTCAAGAATCTATTCC
eEF2_F3	PfeEF2; PF3D7_1451100	Sequencing primer	CATGTTGTTTAGTTGGTGTAG
eEF2_F4	PfeEF2; PF3D7_1451100	Sequencing primer	TGAAGAAAACATGAGAGGTATAG
eEF2_R3	PfeEF2; PF3D7_1451100	Sequencing primer	CTCTTGTTTTTGGATCATCT
eEF2_R4	PfeEF2; PF3D7_1451100	Sequencing primer	CTTATCAGATGTAGGAACCATTT
eEF2_R5	PfeEF2; PF3D7_1451100	Sequencing primer	CTTGTAAACCAGAACCAAAAGAT

KRS_F1	PfKRS; PF3D7_1350100	Forward PCR primer	GATTCTTATGACAAGTAAGTC
KRS_R1	PfKRS; PF3D7_1350100	Reverse PCR primer	CTGGTCGCATAGTGG
KRS_F1seq	PfKRS; PF3D7_1350100	Sequencing primer	CTTCTCACAATTAGTATATC
KRS_R3seq	PfKRS; PF3D7_1350100	Sequencing primer	TAGGATAAGGTGGTGTG
KRS_F2seq	PfKRS; PF3D7_1350100	Sequencing primer	GTTAATAGTAGGTGGTATAG
KRS_R2seq	PfKRS; PF3D7_1350100	Sequencing primer	AAGTAAATTCAGGATTATGTG
KRS_R1seq	PfKRS; PF3D7_1350100	sequencing primer	TCCTTATTACTTGCTTGC
FRS_F1-F1	PfFRS; PF3D7_0109800	Forward PCR primer	GAGTACGAATAATGTGGAAG
FRS_R1	PfFRS; PF3D7_0109800	Reverse PCR primer	ccattaatataaaTCATACAACAC
FRS_R1seq	PfFRS; PF3D7_0109800	Sequencing primer	GGGCAAATAATAGTTCTTCATC
FRS_F1seq	PfFRS; PF3D7_0109800	Sequencing primer	GTACGATACAATTAAATAAAGCTG
FRS_F2seq	PfFRS; PF3D7_0109800	Sequencing primer	CCACAACAACATCCAAGTAG
PheRS-R2seq	PfFRS; PF3D7_0109800	Sequencing primer	GCTGTAGTATGAGTTC
PfPMX_F1	PfPMX; PF3D7_0808200	Forward PCR primer	atcggttataaagacagaaATG
PfPMX_R1	PfPMX; PF3D7_0808200	Reverse PCR primer	TGCTCTTGCTACTCCAAC
PfPMX_F1seq	PfPMX; PF3D7_0808200	Sequencing primer	GTTAGGAAAGCACCTAGTAAG
PfPMX_R1seq	PfPMX; PF3D7_0808200	Sequencing primer	CAGCTGATAACATTCCTG
PfPMX_R2seq	PfPMX; PF3D7_0808200	Sequencing primer	GCATCTTGTTCATACACATG
PfPARE_F1	PfPARE; PF3D7_0709700	Forward PCR primer	gATGAAGAGCCAGGGTGGAGGG
PfPARE_R1	PfPARE; PF3D7_0709700	Reverse PCR primer	TACTTGTTCTTCTTGTTTGGG
PfPARE_F1seq	PfPARE; PF3D7_0709700	Sequencing primer	GAGTTAGCAACGAAACCAT
PfPARE_R1seq	PfPARE; PF3D7_0709700	Sequencing primer	CCATAATATCATTCATATGTG

PfUba2_F1_F1	PfUBA2; Pf3D7_123700	PCR primer for fragment 1	atcctttttgtttgtgtgtc
PfUba2_F1_R1	PfUBA2; Pf3D7_123700	PCR primer for fragment 1	CGATAACATGAGTACATTGTC
PfUba2_F2_F1	PfUBA2; Pf3D7_123700	PCR primer for fragment 2	CAACTATTCCTATACCAATAC
PfUba2_F2_R1	PfUBA2; Pf3D7_123700	PCR primer for fragment 2	GATTATCACTTTCTTCGTCG
PfUba2_F1_R1 seq	PfUBA2; Pf3D7_123700	Sequencing primer	GATTATGTTCACTTTCATCTTC
PfUba2_F1_F1 seq	PfUBA2; Pf3D7_123700	Sequencing primer	CATATAGAAGAAGAATCTAAG
PfUba2_F2_F2 seq	PfUBA2; Pf3D7_123700	Sequencing primer	GAGACATATATTATCCGAC
PfUba2_F2_F1 seq	PfUBA2; Pf3D7_123700	Sequencing primer	CAAGAGGAAACTTAGTTAATG
PfUba2_F2_R1 seq	PfUBA2; Pf3D7_123700	Sequencing primer	CAATTCATTCATACATATATCC
VTHA_mRNA_ F1-F1	PfVATPase-D; PF3D7_1341900	Forward PCR primer	gaatttattttattttttttaaataATGG
VTHA_mRNA_ F1-R3	PfVATPase-D; PF3D7_1341900	Reverse PCR primer	GTACAGGCGTAATTATATTGTC
VTHA_mRNA_ F1-R1seq	PfVATPase-D; PF3D7_1341900	Sequencing primer	GACCTCCTGCTGCAACAC
VTHA_mRNA_ F1-F1seq	PfVATPase-D; PF3D7_1341900	Sequencing primer	GTTGTAACTTTATCCTTATC
